# Prevalence of acetabular labral tears in asymptomatic children

**DOI:** 10.1007/s11832-016-0717-9

**Published:** 2016-02-24

**Authors:** Andrew G. Georgiadis, Mark A. Seeley, Nancy A. Chauvin, Wudbhav N. Sankar

**Affiliations:** Division of Orthopaedic Surgery, The Children’s Hospital of Philadelphia, 34th and Civic Center Blvd, Philadelphia, PA 19104 USA; Division of Radiology, The Children’s Hospital of Philadelphia, Philadelphia, PA USA

**Keywords:** Acetabular labral tears, Pediatric acetabular labrum, MRI labrum

## Abstract

**Purpose:**

Magnetic resonance imaging (MRI) is a sensitive, non-invasive modality to diagnose acetabular labral pathology, and the normal variants of the acetabular labrum have been characterized in adults. However, the prevalence of labral pathology in the asymptomatic pediatric population is unknown.

**Methods:**

All pelvic MRIs performed at a large tertiary-care children’s hospital were reviewed during one calendar year (2014). Only patients aged between 2 and 18 years were included, and scans were excluded for hip pain/pathology or technical inadequacy. A blinded pediatric musculoskeletal radiologist read all eligible scans for the presence or absence of a labral tear.

**Results:**

Three hundred and ninety-four pelvic MRIs were screened, and patients were excluded for hip pain/pathology (85 subjects), or technical inadequacy (190 subjects). One hundred and eight subjects (216 hips) met the inclusion criteria and were technically adequate for analysis. Labral tears were visualized in three of 216 (1.4 %) hips (two of the 110 subjects; 1.9 %).

**Conclusions:**

There is a low rate of asymptomatic labral pathology by MRI in pediatric patients. The clinical history remains the means of differentiating real labral pathology from spurious imaging findings.

**Level of evidence IV:**

Case series (prevalence).

## Introduction

The acetabular labrum is a fibrocartilaginous structure surrounding and adherent to the acetabular rim. It provides concentricity, stability, shock absorption, load distribution, lubrication, and hydrostatic pressurization to the hip joint. Labral tears can alter femoroacetabular joint mechanics and predispose to chondral injury and degenerative osteoarthritis [1, 2].

The gold standard for visualization of labral pathology remains direct open or arthroscopic visualization. The most sensitive and specific imaging modality for evaluation of labral tears is magnetic resonance imaging (MRI), which provides excellent soft-tissue resolution and discrimination of the labrum, chondral surfaces, capsule, joint space, and osseous anatomy [3]. MR arthrography is commonly performed, with pathology identified by intra-articular contrast at the acetabular–labral junction or labral intrasubstance. However, optimized non-contrast protocols have been described with excellent sensitivity in the identification of acetabular labral tears [3–5].

Anatomical variants of the acetabular labrum have been described on MRI [4, 6–10] and the rate of asymptomatic labral pathology on MRI has been established in adult patients [5, 11, 12]. Studies of patients with asymptomatic labral tears all include cohorts of active volunteers, primarily young adults, but no series describes the rates of asymptomatic labral tears detected by MRI among pediatric patients. An estimated 45 million children aged between 6 and 18 years are involved in organized sports in the United States alone [13], and labral tears constitute the operative indication for the majority of pediatric hip arthroscopies [14, 15]. With the backdrop of this increasingly common diagnosis, we sought to establish rates of MRI-based acetabular labral tears in low-demand, asymptomatic children.

## Materials and methods

The Institutional Review Board approved this investigation. A pilot study was performed in which all MRIs of the hip and pelvis performed between January 1, 2014 and December 31, 2014 were queried from radiology records at a large tertiary referral children’s hospital. Searches were performed using a proprietary software package (Illuminate, iSite Radiology v3.5; Koninklijke Philips N.V., Amsterdam, Netherlands), which allows a direct query of radiological studies and the text of their reports. Twenty patients were randomly selected from each list (hip and pelvis). Patient charts were reviewed for underlying hip pathology or diagnosis. Hip pain or an underlying hip disorder was the indication for imaging in all unilateral hip MRIs, compared to 9 of 20 pelvic MRIs. Based on the results of the pilot study and the previously published technical adequacy of pelvic MRI for diagnosing labral pathology [12], pelvic MRI was selected as the imaging sequence of choice for examining asymptomatic patients. The pilot study images were reviewed by a pediatric musculoskeletal radiologist (NAC) and the senior author (WNS), who agreed upon minimum technical criteria for diagnosing a labral tear using a non-optimized MRI protocol.

**Fig. 1:**
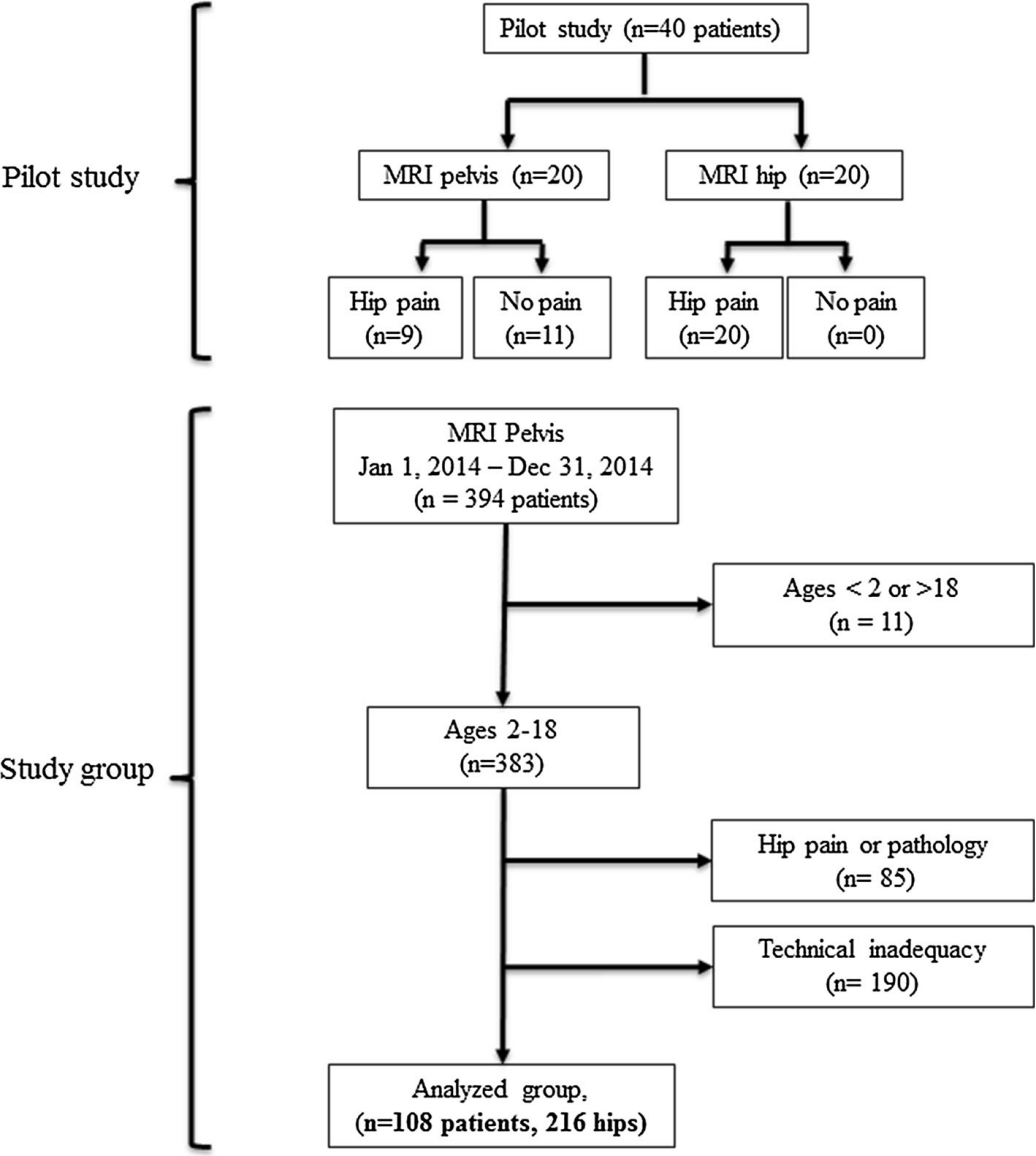
Flow diagram detailing the pilot study establishing pelvic MRI for the study protocol, and establishment of the study group after exclusions

All MRIs of the pelvis performed at a tertiary referral children’s hospital between January 1, 2014 and December 31, 2014 were compiled and reviewed (Fig. 1). Patients aged 2–18 years were included and their medical charts were reviewed for pertinent clinical history and study indications. Subjects were excluded if there was a history of unilateral hip pathology. MRIs met inclusion criteria if there were fluid-sensitive sequences in at least 2 imaging planes, complete acetabular coverage, and image slice thickness of <4 mm. Studies were excluded if there was patient motion artifact or poor spatial resolution due to suboptimal field of view or slice thickness for age, precluding evaluation of the labrum. Each hip was considered unique and independent. After exclusions, a fellowship-trained pediatric radiologist with expertise in musculoskeletal imaging reviewed each MRI of the pelvis for labral tears or labral pathology. The radiologist (NAC) was blinded to the results of the original report. Previously described normal anatomic variants were not considered labral tears (e.g., intralabral signal intensity, or rounded or irregular borders) [7–9]. A priori, paralabral cysts were considered labral tears even if a tear was not directly visualized. All identified tears were re-reviewed by another senior radiologist with additional expertise in pediatric musculoskeletal imaging to confirm the presence of the tear.

All scans were performed on one of eight MRI machines within the same tertiary referral system of a large children’s hospital. Six were 3T systems—five MAGNETOM^®^ Avanto, Verio, Trio, and Skyra (Siemens, Munich, Germany) and one GE Discovery 750w (GE Healthcare, Little Chalfont, UK). Two were 1.5T systems, both MAGNETOM^®^ Avanto (Siemens, Munich, Germany).

## Results

**Fig. 2:**
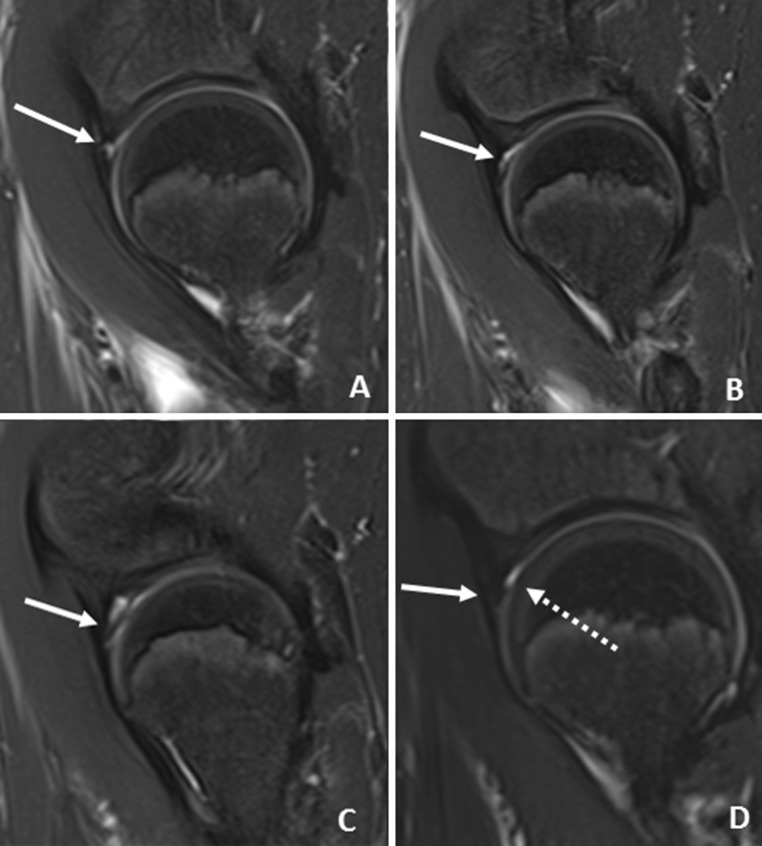
Consecutive sagittal T2-weighted fat-saturated images of an 11-year-old boy who underwent MRI of the pelvis for possible inflammatory bowel disease. **a** *Image* of the right hip demonstrates abnormal fluid signal extending along the base of the labrum at the labral–cartilaginous junction (*arrow*). **b** There is an abnormal thin band of fluid signal or cleft extending along the anterosuperior aspect of the labrum consistent with tear. **c** Abnormal intrasubstance signal is noted within the labrum (*arrow*). **d** Normal appearance of the left hip and labrum (*solid arrow*). A trace amount of physiologic fluid is noted (*dashed arrow*)

Three hundred and ninety-four unique pelvic MRIs were performed at the study institution during the calendar year 2014. After exclusions for hip pain or hip pathology (85 subjects), or technical inadequacy (190 subjects), 108 subjects (216 hips) were available for analysis. The mean subject age was 11.9 years (range 2–18 years, Fig. 2).

The indications for pelvic imaging in the study group included gastrointestinal disease (21 patients), abdominal/pelvic malignancy or surveillance (38 patients), genitourinary disease (13 patients), infection or other malignancy (28 patients), rule-out sacroiliitis for rheumatologic disease (9 patients), and neurogenic symptoms (1 patient).

**Fig. 3:**
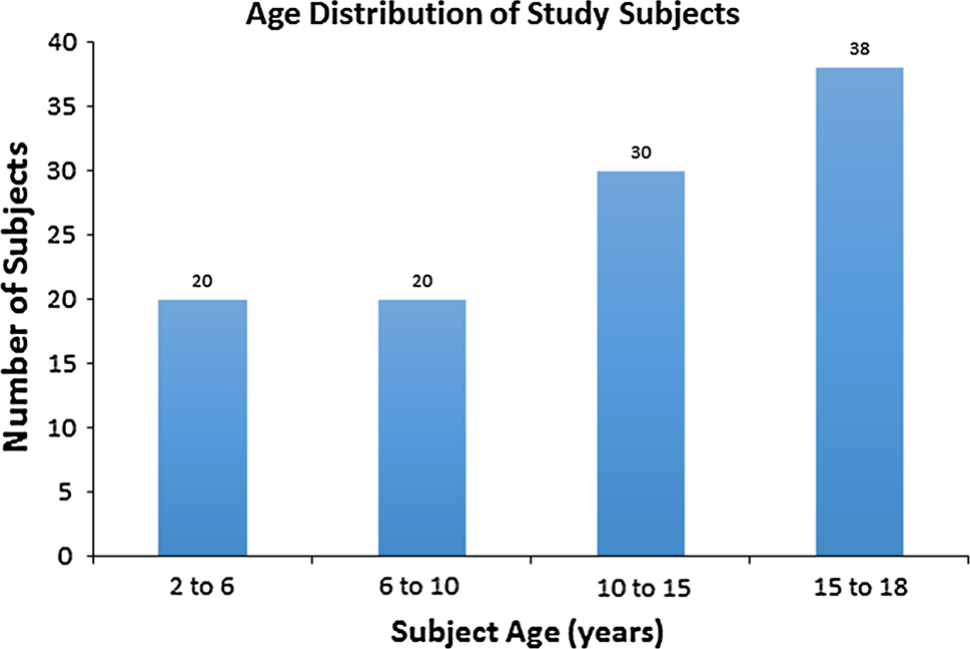
Age distribution of asymptomatic patients undergoing MRI of the pelvis after exclusions for technical inadequacy

Three labral tears (3/216 hips, 1.4 %) in two patients (2/108, 1.9 %) were identified. All were confirmed to be present by a second experienced pediatric musculoskeletal radiologist. One patient was an 11.7-year-old male with recurrent *Clostridium difficile* colitis undergoing an MRI to rule out inflammatory bowel changes, who had a right labral tear (Fig. 3). The other patient was a 9.4 year-old female with bilateral labral tears diagnosed by a postsurgical MRI obtained after sacrococcygeal teratoma resection. All were considered complete tears, defined as complete separation of at the chondrolabral junction. No tears were identified on the index read by the original radiologist at the time of study performance.

## Discussion

The acetabular labrum is a fibrocartilaginous ring between the rim of the bony acetabulum and the hip joint capsule. It is triangular in cross-section and functions to support shock absorption and load dispersal, maintain synovial fluid dynamics, and extend the concentricity of the femoroacetabular articulation. Physical disruption of the labrum can alter hip joint mechanics, leading to abnormal loading, chondral injury, and eventually degenerative osteoarthritis [1, 2]. The anterosuperior labrum has a lower elastic modulus which is a suspected mechanical reason for labral tears in this area [16].

The MRI appearance of the pediatric labrum has been observed to decrease in size relative to the femoral head during childhood, with a characteristic triangular appearance in the anterosuperior weight-bearing region and flattening posterosuperiorly [17]. Abe et al. described the morphological variants of the labrum, finding the free apex to be triangular or rounded in young adults, whereas adults aged >40 years had a 20 % rate of irregular or absent labra [6]. Other authors found a high-intensity signal within the weight-bearing portion to be a normal variant, and with increasing age found increasing intralabral intensity changes that communicated with the hip joint [7, 9]. More confounding still, 15 % of all labra can be morphologically asymmetrical in the same patient [7]. Some authors contend that asymptomatic labral tears do not constitute ‘tears’ or ‘lesions’ and are clinically insignificant [18].

There is an increasing burden of femoroacetabular impingement (FAI) surgery performed on young people, particularly as arthroscopic techniques develop. In a large epidemiological study, the largest number of patients undergoing FAI surgery were aged between 10 and 19 years, and >90 % had labral pathology at the time of surgical treatment [19]. Labral injury is also increasingly appreciated in open surgery. In another study, 88 % of patients undergoing periacetabular osteotomy had labral tears (average age 21 years) [20], as did those undergoing surgical hip dislocations for FAI (26/26 of those with ‘cam’ and 5/16 with ‘pincer’ lesions) [21]. Establishing the rate of labral tears in asymptomatic pediatric populations will become increasingly important as modern MRI continues to improve and surgery on the labra of young people becomes more common.

Four studies have included small numbers of adolescents in the description of labral variants, although granular subgroup results were not reported [6–9]. In 1996, Lecouvet et al. [9] performed 0.5-T and 1.5-T MRIs of asymptomatic hips in volunteers aged 15–82 years, finding 13 % of hips in those aged 15–24 years had high-signal intensity communicating with the joint, which also significantly increased with age. In a 1.5-T study among an asymptomatic group of 71 patients aged 13–65 years, Abe et al. [6] found 3 % of ‘labral’ segments in the 10- to 19-year age group contained high-signal intensity communicating with the articular surface. Aydingoz et al. [7] performed an 0.5-T study looking at 360 labra in 180 asymptomatic patients aged 10–50+ years. Only one patient (3.6 %) in the 10- to 19-year-old cohort had intralabral intensity increases on T2-weighted non-contrast images.

All published investigations of labral ‘tears’ in asymptomatic volunteers have been non-contrast studies in young adults with high-activity demands (Table 1). A study of male Swiss military recruits (average age 19.9 years, patients <18 years excluded) found that 71.7 % (175/244 hips) had labral tears by hip MRI, with higher rates in those with femoral ‘cam’ lesions [11]. Silvis et al. [12] performed both 3.0-T hip and pelvic examinations of collegiate and professional hockey players, although ages were not reported, and 22 of 78 hips had a labral tear (28 %). In an optimized 1.5-T study of asymptomatic active-duty Air Force members in the United States (aged 27–43 years), >80 % had acetabular labral tears [5]. These results are comparable to the 30 % false positive rate of MRI in the diagnosis of meniscal tears in the knee [22]. Lee et al. [23] found an asymptomatic labral tear rate of 38.6 % in non-arthrogram MR scans of 19- to 41-year-old volunteers.

**Table 1:** Studies of asymptomatic labral tear rates by non-contrast MRI protocols

References	Pts	Hips	Labral tears *N* (% of hips)	Age (years)	Field strength	Notes
Reichenbach et al. [11]	244	244	175 (72)	19.9	1.5-T	Swiss military recruits, excluded <18 years
Silvis et al. [12]	39	78	22 (28)	NR	3.0-T	College/pro hockey players, used both pelvic and bilateral hip MRI
Schmitz et al. [5]	42	42	34 (81)	27–42	1.5-T	Active-duty Air Force volunteers
Lee et al. [23]	70	140	27 (19.2)	19–41	3.0-T	Medical student and allied health professional volunteers

NB: all studies include young adults but no pediatric patients

*NR* Not reported

Our study found a very low rate of asymptomatic labral tears in a low-demand pediatric cohort. Our study design was strengthened by modern, high-strength magnets that compare favorably to those used in original descriptions of labral morphology [24]. Technological improvements are in clear evidence when older studies in adult volunteers reported 8 % asymptomatic labral tear rates [8], whereas studies with modern coils report up to 80 % [5]. Our pelvic protocols were similar to the newest non-contrast protocols in resolution, field-of-view, and slice thickness and were able to provide enhancement of joint fluid in many cases [3, 4, 12]. The accuracy of hip MRI has been shown to be dependent on radiologist practice settings [25] and all scans in our study were evaluated by a pediatric musculoskeletal radiologist at a tertiary academic children’s hospital. To date, the only investigations of asymptomatic teenagers have been included in the results of adults, and all studies included high-level athletes with clear mechanisms by which asymptomatic hip trauma might have been incurred [5, 11, 12]. Furthermore, as this study involved purely asymptomatic patients whose indications for imaging consisted of chronic medical conditions, they likely had lower physical demands. Inexorable technological advances will improve our understanding of which labral tears are real and which are artifactual. There are preliminary feasibility results of 7-T magnets used in simultaneous hip MRI [26], and 21.1-T imaging has been used in recent animal studies [27].

Our findings should be interpreted in the context of several limitations. There were technical constraints to scan methodology, as all studies were performed for unrelated pelvic or abdominal indications. Thus, there were no routine axial oblique images acquired that would optimize anterolateral labral visualization. We attempted to mitigate this weakness by adhering to strict minimums of slice thickness, adequate fields of view, and including those studies with axial, sagittal, and coronal images. However, pelvic sequences that include both hips reduce the resolution of available images, and the evaluation of hip abnormalities is inferior to that for groin pathology [12]. We did not assess proximal femoral or acetabular morphology because of the heterogeneity of skeletal maturity among our patients. Bony deformity of the hip has been correlated with labral pathology [28], although this has been contested [29]. Our examinations did not include intra-articular contrast which has been shown to increase sensitivity of depicting labral abnormalities in hip [30] and pelvic MRI [12]. Ultimately, there are ethical and technical constraints to evaluating pediatric patients, as volunteer magnetic resonance arthrograms are not feasible in asymptomatic children, hence our study design.

Optimized non-contrast scans are often used to investigate labral pathology. These results stress that any MRI finding should be used to corroborate the clinical diagnosis. It is likely that low-demand, asymptomatic pediatric patients have truly low rates of labral pathology on MRI, and certainly lower rates than young adult athletes. The clinical history remains integral in differentiating spurious imaging results from those with clinical significance.
